# Supplementary material for: Embryonic environment and transgenerational effects in quail
Source: Genet Sel Evol. 2017 Jan 26;49:14. doi: 10.1186/s12711-017-0292-7 (PMC5270212; doi:10.1186/s12711-017-0292-7)
Supplement: Supplementary file 1 — Additional file 1: Figure S1. Schematic representation of the mirror mating design. As an example, the figure shows the pedigree of two Epi+ and Epi− birds from the G3 generation (9E− and 9E+) starting from their four founding single-pair matings (SPM) at generation G0. Lines of descent are identified with a different color from each founding SPM. Transmission arrows have solid lines for Epi+ birds and broken ones for Epi− birds. Homologous birds 9E+ and 9E− have mirror-like, parallel, pedigrees with the same expected genetic contribution from their founders. In this study, matings were designed so that only homologous birds were obtained in the G3 generation. The 8E+ and 8E− parents are produced the same way as the 7E+ and 7E−, from 4 different founder pairs. [file 12711_2017_292_MOESM1_ESM.pptx]

## Slide 1
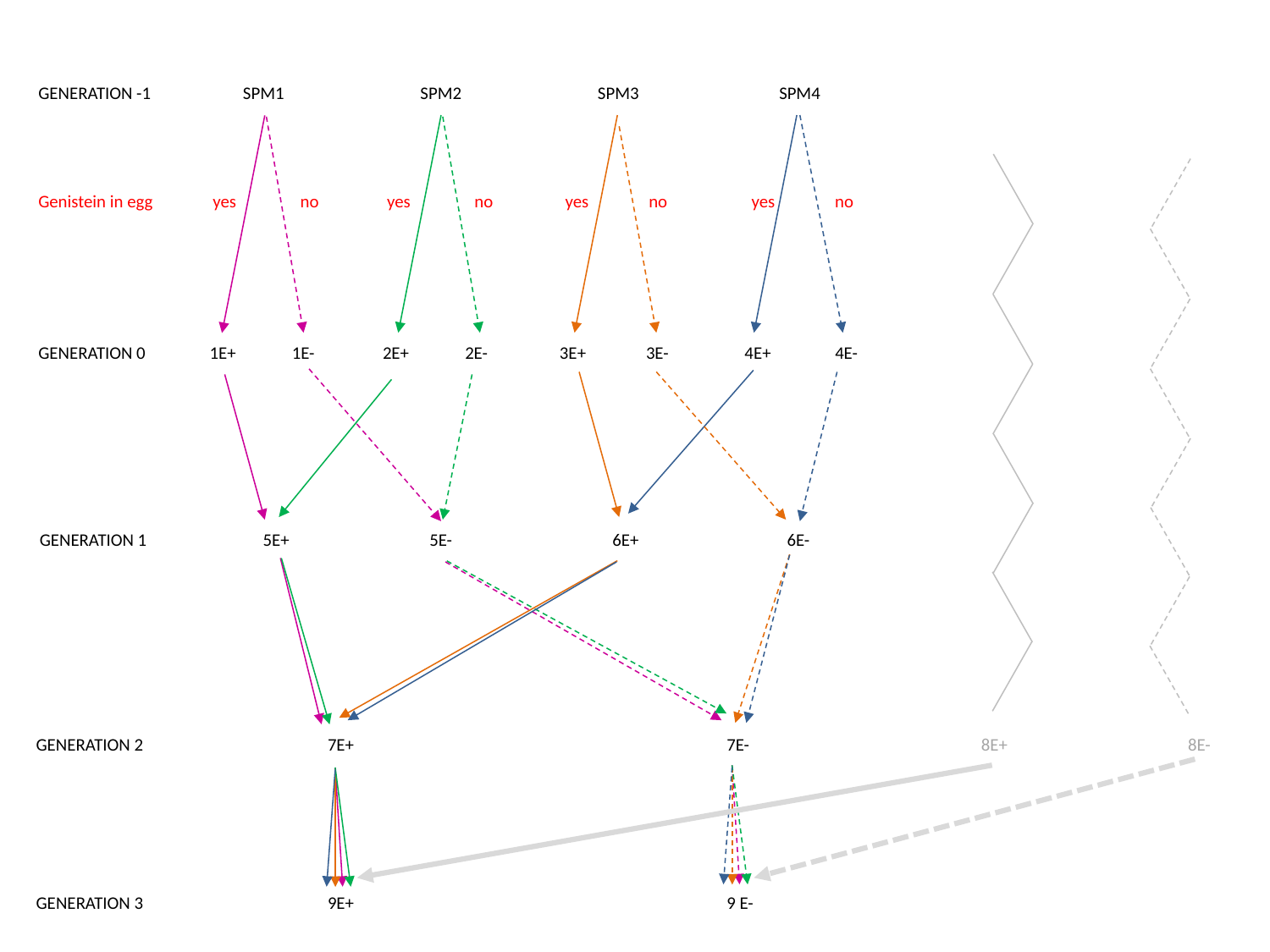

GENERATION -1 SPM1 SPM2 SPM3 SPM4
Genistein in egg yes no yes no yes no yes no
GENERATION 0 1E+ 1E- 2E+ 2E- 3E+ 3E- 4E+ 4E-
GENERATION 1 5E+ 5E- 6E+ 6E-
GENERATION 2 7E+ 7E-		 8E+ 8E-
GENERATION 3 9E+ 9 E-
