# Supplementary figures and images for: Embryonic environment and transgenerational effects in quail
Source: Genet Sel Evol. 2017 Jan 26;49:14. doi: 10.1186/s12711-017-0292-7 (PMC5270212; doi:10.1186/s12711-017-0292-7)

## Slide 1
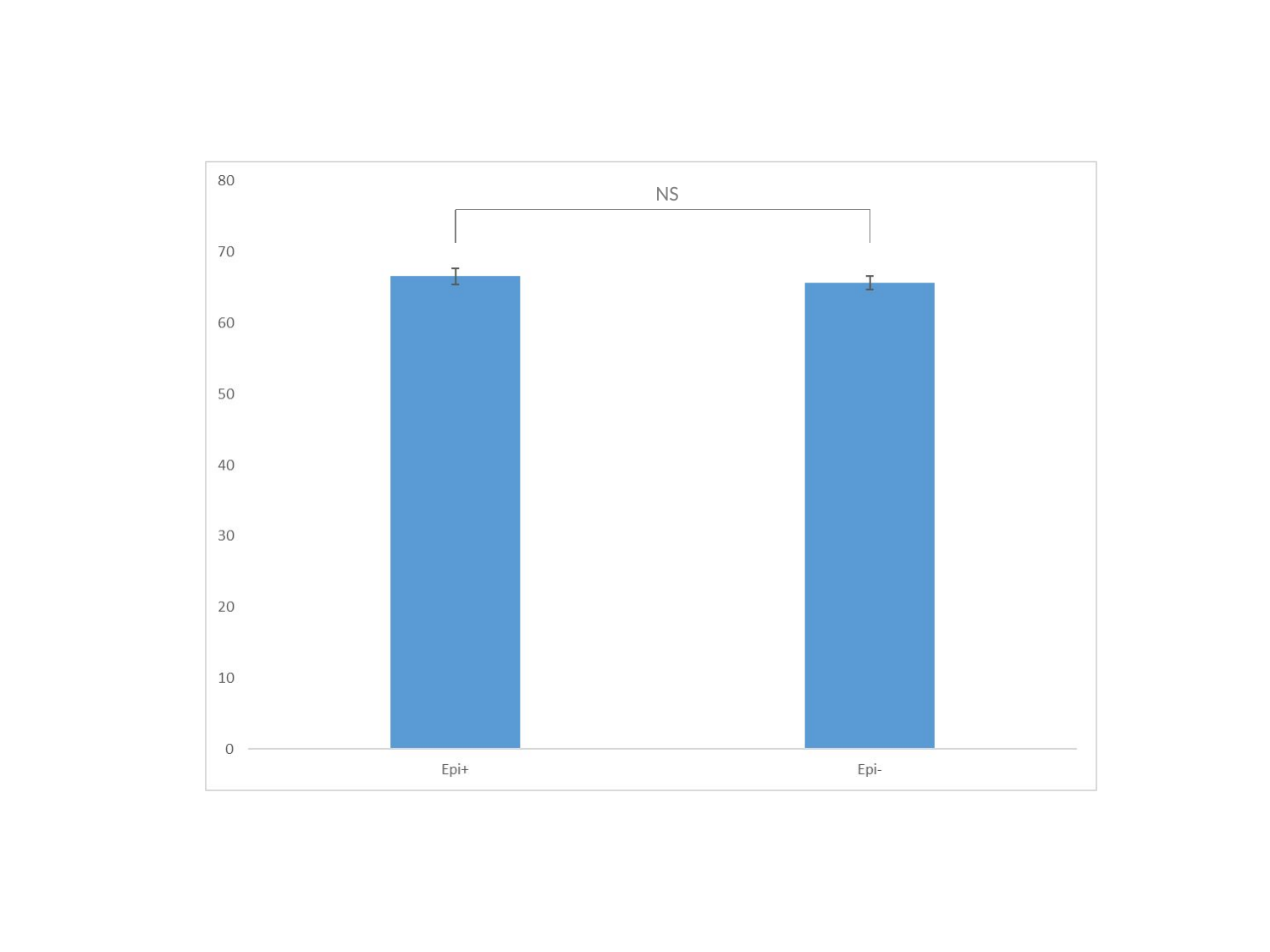

NS

Supplement: Supplementary file 3 — Additional file 3. LUMA results on G3 individuals. Figure S2 shows mean and standard-deviation of the methylation level (%). No significant difference was observed between the lines. [file 12711_2017_292_MOESM3_ESM.pptx]
